# Odontogenic cervico-facial cellulitis during pregnancy: about 3 cases

**DOI:** 10.11604/pamj.2020.36.258.24864

**Published:** 2020-08-10

**Authors:** Zakaria Aziz, Salma Aboulouidad, Mohammed El Bouihi, Saad Fawzi, Mohammed Lakouichmi, Nadia Mansouri Hattab

**Affiliations:** 1 Maxillo Facial Surgery Department, University Hospital Center Mohammed VI, Marrakech, Morocco,; 2 Maxillo Facial Surgery Department, Avicenne Military Hospital, Cadi Ayyad University, Marrakech, Morocco

**Keywords:** Facial cellulitis, odontogenic, pregnancy, extraction

## Abstract

Pregnancy is considered as a risk factor for development, severity, and complications of odontogenic infections. Without adequate treatment, the infection can spread and threaten both the mother’s and the foetus lives. We aim to analyze the predisposing factors, diagnostic and therapeutic aspects of cervico-facial cellulitis during pregnancy, through a descriptive retrospective study conducted at oral and maxillofacial surgery department of Mohamed VI university hospital center at Marrakesh, between June 2017 and June 2019. A total of three patients; all patients were at their last trimester were recruited. Every patient was immediately given intravenous antibiotics, drainage was carried out under local anesthesia, and the causing tooth was removed. During hospitalization, one patient was referred to the gynaecology department for preterm labor, while the remaining two patients were discharged after the pus drainage has stopped. The possible compromise of oral health during pregnancy is well known, however severe odontogenic infections are rarely considered in the literature. It is essential to aggressively treat the gravid patient to minimize the risk of infection spreading to the facial spaces. Moreover, poor oral health in pregnancy has been implicated in adverse birth outcomes, specifically prematurity. We recommend upgrading communication between obstetrician and dentists so that regular routine dental visits are planned for pregnant patients during early stages of pregnancy in order to identify and manage the problem as early as possible.

## Introduction

Odontogenic infection is the most prevalent disease worldwide and it´s known to be the main cause of facial space infections [1]. Without adequate treatment, the infection can spread along fascial planes caudally to the cranial base, and in a rostral direction to the mediastinum [2]. On the other hand, pregnancy is an altered physiological state with decreased immune functions that affects almost every system of the body [3], the implication of which is rapidly spreading cellulitis that can be life threatening for both the mother and the foetus. In fact, pregnancy is considered as a risk factor for development, severity, and complications of odontogenic infections [4, 5]. An early diagnosis based on clinical features is crucial for an effective therapy. Management plan should be such that it should maximize benefit to the mother and minimizing the risk to the developing fetus [6]. Consequently, treatment decision should be taken by a multidisciplinary team including the obstetrician, the oral and maxillofacial surgeon and the anesthesiologist [2]. The aim of this paper was to analyze the predisposing factors, diagnostic and therapeutic aspects of cervico-facial cellulitis during pregnancy.

## Methods

We conducted a descriptive retrospective study at oral and maxillofacial surgery department, Mohamed VI university hospital of Marrakesh, between June 2017 and June 2019 including all pregnant women presenting with odontogenic facial cellulitis. The following variables were studied: patient age, gestational age, time from onset of symptoms, previous medication, extra and intraoral examination, involved tooth and radiographic assessments, duration of hospital stay. All the patients were hospitalized and gynaecological opinion was taken for both maternal and fetal health status. They underwent blood tests including complete blood count and CRP as inflammatory parameter. Drainage was carried out under local anesthesia using a N°15 blade then a rubber drain was placed in the cavity and maintained till stoppage of the pus drainage. Once microbiological sample was taken, the patient was immediately given intravenous antibiotics (Amoxicillin+ clavulanic acid 1g/8 hours) and a bolus corticotherapy (120 mg methyprednisolone). The causing tooth was removed as soon as mouth opening has improved.

## Results

**Figure 1:**
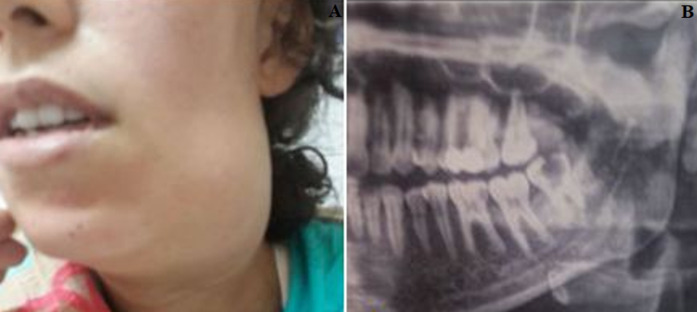
A) picture shoswing extensive submandibular space infection resulting in facial swelling and limited mouth opening; B) orthopantomogram of the same patient showing a carious third molar (N°38)

A total of three patients were recruited in this work (Table 1). The mean age was 27.5 years and all patients were at their last trimester (mean gestational age at 31 weeks). Investigations revealed taking non-steroidal anti-inflammatory drugs to relief a toothache. The duration of symptoms ranged from 5 to 9 days. Clinically, they all presented with limited mouth opening and facial swelling (Figure 1). Tenderness and warmth were observed on palpation extraorally and the offending teeth were tender to percussion. Laboratory investigations revealed increased CRP and leukocyte count. Staphylococcus aureus was found in one microbiological screening while the others showed sterile culture. The mean hospital stay duration was 12.6 days and switch to oral antibiotics was done once local and systemic infection signs disappeared. The total duration of antibiotherapy was 21 days. During hospitalization, one patient was referred to the gynaecology department for preterm labor at 35 weeks giving birth to a healthy male newborn. The remaining two patients were discharged after the pus drainage has stopped.

**Table 1:** summary of patient´s data

N°	Age	Gestational age	Duration of symptoms	Involvement space	Causing tooth	Leukocyte count	CRP	Admission duration	Condition on discharge
1	31	29	7 days	Submandibular	N°38	10 500 C/mm^3^	29 mg	13 days	stable
2	24	35	9 days	submandibular	N°48	13 600 C/mm^3^	36 mg	17 days	Preterm delivery
3	28	30	5 days	Submental	N°32	11 500 C/mm^3^	15 mg	8 days	Stable

## Discussion

Cervico-facial cellulitis are bacterial infections affecting the cellular adipose spaces of the head and the neck. They are a local-regional complication that is most of the time of dental origin [7]. Odontogenic infections can lead to serious complications if not treated early, including upper airway obstruction, descending mediastinitis, septic shock, acute renal failure, disseminated intravascular coagulation, jugular vein thrombosis, carotid artery pseudoaneurysm, and pericardial effusion [8]. The possible compromise of oral health during pregnancy is well known, however severe odontogenic infections are rarely considered in the literature [1]. Moreover, poor oral health in pregnancy has been implicated in adverse birth outcomes, specifically prematurity, development of preeclampsia, and infants born at small-for-gestational-age [9]. Pregnancy is associated with many physiological and hormonal changes, providing the oral and maxillofacial surgeon with many challenges [10]. This change place the mother at a higher risk of infection or of doing worse, once infected. The immune response is greatly diminished during pregnancy, resulting in potential faster progression of an infection. In addition, there is decreased neutrophil chemotaxis, cell-mediated immunity, and natural killer cell activity [11, 12]. From an oral perspective, pregnancy-associated hormonal changes also affect the gingival tissues. They become much more sensitive and susceptible to irritation from soft plaque. Plaque accumulates, becomes hard calculus deposits on the teeth, and harbors bacteria in large numbers resulting in a constant, low-grade intraoral infection. An exaggerated local inflammatory response can then begin and may result in erythematous and edematous swelling of the gingiva between the teeth, also known as pregnancy gingivitis [13]. Approximately 70% pregnant women have this condition, even with routine oral care [10]. Furthermore, during pregnancy, women tend to have frequent meals and snacks in addition to increased acid reflux and vomiting, which cause further accumulation of plaque, as well as an increase in decay or rapid progression of previously present decay [13, 14].

These changes are aggravated during second and last trimester of pregnancy [10]. As in the present study where all patients were at their last trimester. On the other hand, oral health procedures in pregnancy are often avoided and misunderstood by physicians, dentists, and patients. There was some degree of confusion over the safety of accessing dental care during pregnancy [15]. In fact, none of our patients sought for dental care before or during pregnancy, as in Elneel Ahmed Mohamed Ali study [15] where 50% of patients had experienced toothaches many months before but didn´t seek any treatment because of misconceptions among women regarding dental treatment during pregnancy and 40% patients were delayed because of instructions from their doctors. Authors recommended that dental care should be provided during the second trimester so as to reduce any risk to the early development of the fetus and for the woman´s comfort [5, 12, 15]. The diagnosis of facial cellulitis is usually obvious based on clinical grounds: facial and cervical painful swelling sometimes associated to pus discharge [16]. If not, a radiograph and a CT scan is performed [1]. The prevalence of odontogenic infection involving fascial spaces in descending order is submandibular, submental, buccal and sublingual [15, 17, 18], this findings are consistent with our results. There is no contraindication to the sparing use of radiology. It has been shown that doses of less than 5 to 10 centigrays (cGy) have no association with increased development of congenital defects or intra-uterine growth retardation [10]. In general, a single orthopantomogram will provide sufficient information at an acceptable radiation exposure. With advanced spreading odontogenic infections into the neck, generally this is best demonstrated by a CT scan. A single CT scan has less than the normal safe level of irradiation (e.g. 5-10 cGy) but is greater than for an Orthopantomogram. Thus, CT scanning is best avoided in pregnant patients and only used if strongly clinically indicated, such as to define a pus collection in patients not responding to surgical management. Ultrasound has a place in defining moderate to large pus collections in the neck and it should be considered over and above a CT scan [19].

In our study, all patients had orthopantomograms that helped defining the causing tooth and the most commonly involved tooth was mandibular last molar. Management of mild infections should be managed via incision/ drainage under local anesthetic with subsequent antibiotic coverage. It is essential to aggressively treat the gravid patient to minimize the risk of infection spreading to the facial spaces. Facial space infection should be handled in a standard fashion: airway assessment (if any doubt, intubate), imaging (computed tomography scan), and to the operating room for adequate incision and drainage. Postoperatively, if the patient is unable to maintain oral intake, parenteral nutritional support must be instituted. More severe infections should be managed in the operating room under general anesthesia with intravenous antibiotics and incision and drainage [1]. Antibiotics that are acceptable during pregnancy include penicillin, amoxicillin, and clindamycin. Tetracycline should be avoided since it tends to cause permanent discoloration of primary and temporary dentition of the unborn child [12]. Local anesthesia is preferred than general anesthesia can induce premature delivery, inhalation and pneumonia risk [10, 16]. This paper showed that the pregnant patients with severe odontogenic infections were successfully managed with one case of preterm birth. A metanalysis indicates a likely association between preterm birth and paradontal status [20]. Our work serves as a reminder for all health practitioners to not neglect even minimal complaints of dental pain and diagnosis odontogenic infections in pregnancy at an early stage, and then refer these patients for timely and appropriate management by a multi-disciplinary team.

## Conclusion

Odontogenic cellulitis during pregnancy is relatively frequent in our context. This is due to poor oral health, inaccessibility to dental care and lack of sanitary programs including dental consultation. We recommend upgrading communication between obstetrician and dentists so that regular routine dental visits are planned for pregnant patients during early stages of pregnancy in order to identify and manage the problem as early as possible.

### What is known about this topic

- Pregnancy is considered as a risk factor for development of odontogenic infections;
- Cervico-facial cellulitis can be life-threatening for both the mother and the fetus.

### What this study adds

- Cervico facial cellulitis during pregnancy leads to obstetrical complications such as preterm birth;
- Early diagnosis and management strategy of cervico facial cellulitis in pregnant women;
- The importance of dental care in pregnant women.
